# Novel Anthropometry Based on 3D-Bodyscans Applied to a Large Population Based Cohort

**DOI:** 10.1371/journal.pone.0159887

**Published:** 2016-07-28

**Authors:** Henry Löffler-Wirth, Edith Willscher, Peter Ahnert, Kerstin Wirkner, Christoph Engel, Markus Loeffler, Hans Binder

**Affiliations:** 1 Interdisciplinary Centre for Bioinformatics, Leipzig University, Härtelstraße 16 – 18, 04107 Leipzig, Germany; 2 LIFE, Leipzig Research Center for Civilization Diseases; Leipzig University, Philipp-Rosenthal-Straße 27, 04103 Leipzig, Germany; 3 Institute for Medical Informatics, Statistics and Epidemiology, Leipzig University, Härtelstraße 16 – 18, 04107 Leipzig, Germany; Seoul National University College of Medicine, REPUBLIC OF KOREA

## Abstract

Three-dimensional (3D) whole body scanners are increasingly used as precise measuring tools for the rapid quantification of anthropometric measures in epidemiological studies. We analyzed 3D whole body scanning data of nearly 10,000 participants of a cohort collected from the adult population of Leipzig, one of the largest cities in Eastern Germany. We present a novel approach for the systematic analysis of this data which aims at identifying distinguishable clusters of body shapes called body types. In the first step, our method aggregates body measures provided by the scanner into meta-measures, each representing one relevant dimension of the body shape. In a next step, we stratified the cohort into body types and assessed their stability and dependence on the size of the underlying cohort. Using self-organizing maps (SOM) we identified thirteen robust meta-measures and fifteen body types comprising between 1 and 18 percent of the total cohort size. Thirteen of them are virtually gender specific (six for women and seven for men) and thus reflect most abundant body shapes of women and men. Two body types include both women and men, and describe androgynous body shapes that lack typical gender specific features. The body types disentangle a large variability of body shapes enabling distinctions which go beyond the traditional indices such as body mass index, the waist-to-height ratio, the waist-to-hip ratio and the mortality-hazard ABSI-index. In a next step, we will link the identified body types with disease predispositions to study how size and shape of the human body impact health and disease.

## Introduction

Anthropometric measures are important to assess developmental normality and predispositions to diseases and to calculate drug and chemotherapy dosages. The relationship between the fat distribution, the associated human body shape and health risk, e.g. for cardiovascular diseases, metabolic syndrome or cancer, are a major issue in many population studies [1–6] where size and shape of the human body have traditionally measured in terms of only a few anthropometric measures. Simple combinations of basal measures such as height, waist circumference, and weight were combined into ‘health indices’ to judge the health status of human individuals. The impact and suitability of health indices such as the BMI (body-mass index [7]), WHtR (waist circumference to height ratio [1]), WHR (waist to hip circumference ratio [8,9]) and ABSI (a body size index [3]) were under discussion in the context of the “obesity-mortality paradox” [10], showing that moderate overweight does not imply shorter lifetime. These results call for a rethinking of how metabolic health is assessed in terms of alternative anthropometric measures which better characterize the relationship between the dimensions of the human body and health.

Currently, whole body scanners using triangulation are the most effective measuring tools for the rapid, accurate, precise and reproducible quantification of the dimensions of the human body [11–16]. These devices capture 3D body models in a few seconds of measurement. Therefore, the participant is illuminated by four lasers which project horizontal lines around the body. Those lines are captured by eight cameras on different heights and utilized to triangulate the body surface, which is then transformed into about one hundred length and circumference measures by appropriate software tools in a fully automated way with high reproducibility, precision and accuracy [16–18]. These new and amended data are expected to improve the diagnostics of many diseases, replacing the current reliance on simple body indices [15,16].

Body scanning produces new types of data which, in turn, need new algorithms and approaches for 3D shape analysis including dimension reduction and normalization [19]. They also challenge new concepts for anthropometric phenotyping to get finer morphological distinctions for whole-body characteristics [20]. The first and, to our best knowledge, so-far unique study of ‘body-‘ typing (i.e. the quantification and clustering of human body shapes) with inputs from 3D anthropometry was published only recently [21]. This first attempt to cluster body scanner data is however based on a relatively small cohort of about 300 adult people. It provided a simple classification into endomorphic (high fatness), ectomorphic (high linearity), and endo-mesomorphic (a mixture of fatness and muscularity) body types. Other studies based on 3D body scanning used only a few single measures to derive combined indices such as BMI or WTH without considering the increased set of body measures potentially available [13,14].

3D body scanning is ideal for screening large populations of subjects in large-scale epidemiological surveys due to detailed acquisition of body dimensions, and easy and efficient use [13]. It is applied to generate a database of human physical dimensions for men and women of various weights, between the ages of 18 and 65 years of a total of 2,500 people in the United States and 2,500 in Europe (The Netherlands and Italy) in the frame of the CAESAR (Civilian American and European Surface Anthropometry Resource Project) project [22], which promoted to tackle a series of methodical issues of 3D scanning technology [17–19].

3D body scans were applied in the Leipzig Research Center for Civilization Diseases (LIFE). LIFE-ADULT (see reference [23] for a description of the study design) so far conducted the largest population based study with an extensive phenotyping of urban individuals in Germany. It has recently completed the baseline examination of 10,000 randomly selected adult participants from Leipzig, a city in eastern Germany with 550,000 inhabitants. The general objective is to investigate prevalences, early onset markers, and the role of lifestyle factors of major civilization diseases. All participants underwent an extensive core assessment program including besides anthropometry structured interviews, questionnaires, physical examinations and biospecimen collection. The study covers a main age range from 40–79 years and predominantly collects people of middle European ethnicity. A follow-up investigation is planned to start from 2016, re-assessing the same participants and allowing longitudinal comparisons.

In this publication we systematically analyzed the complete set of body measures provided by 3D body scanning of 8,499 adult participants of the LIFE study to define novel anthropometric phenotypes with possible relevance for lifestyle factors and civilization diseases. This data constitutes, to our best knowledge, one of the largest sets of such data presently available. We pursue several objectives: How many and particularly what kinds of dimensions of body shape are relevant? Are there characteristic ‘body types’? How to determine, to define and to describe such body types in a robust fashion? For this we present a methodical framework of data analysis using machine learning as the basal clustering technique. We demonstrate that our approach allows for representation of human body shapes with high-resolution. It provides robust body types as an important prerequisite for identifying new anthropometric factors of health risk in subsequent, presently ongoing analyses.

## Material and Methods

### Ethics approval and consent to participate

As a prerequisite to enrolment, written informed consent was obtained from all participants. The study was approved by the responsible institutional ethics board of the Medical Faculty of the University of Leipzig. The data privacy and safety concept of the study was approved by the responsible data protection officer.

### 3D body scanning of the LIFE adult cohort

In this publication we analyzed anthropometric 3D body scanner data collected in the LIFE-ADULT cohort between 8/2011 and 11/2014. A comprehensive description of the study design is given in [23]. The study comprised inter alia interviews, questionnaires, classical anthropometry, laboratory investigations, biobanking, endocrinological, cardiovascular and cognitive assessments, MRI of the brain, and 3D body scanning. Basic anthropometric characteristics of the cohort are summarized in Table 1.

**Table 1:** Basic characteristics of the LIFE-ADULT cohort (mean values ± standard deviation). Distribution functions can be found in S1 File.

Gender	Male	Female
Number of participants	4,117	4,363
Age (y)	57 ± 13	56 ± 12
Height (cm)	176 ± 7	165 ± 7
Weight (kg)	86 ± 14	71 ± 14
Waist circumference (cm)	101 ± 12	91 ± 13
BMI (kg/m^2^)	28 ± 4	26 ± 5
WHR	0.96 ± 0.08	0.84 ± 0.08
WHtR	0.57 ± 0.07	0.55 ± 0.09

3D body scanning was performed by means of a commercial ‘Vitus Smart XXL’ 3D laser scanner (Human Solutions GmbH, Kaiserslautern, Germany) which provides an image of the body surface of each participant. Body measures were extracted from this image using AnthroScan 2.9.9 software (Human Solutions GmbH) and stored in the LIFE research data base. Measurement and data generation are in agreement with ISO 20685, the international standard for 3-D scanning methodologies for internationally compatible anthropometric databases. About 20–30 participants passed the study program of LIFE per day including 3D body scanning. The scanner was calibrated every morning before data collection using a standard calibration body. Measurements of all participants (standing position) were realized in a standardized way by applying in-house standard operating procedures (SOP) based on manufacturer’s instructions. SOP define the positioning of the participants, scanner settings, and the protocol throughout the scanning process. Body measures were extracted automatically by the scanner software using default parameter settings as implemented by the manufacturer which includes Landmark identification and gap filling algorithms. The data of all participants were generated using the same scanner device and the same software as described above. We validated the results of different software versions (v2.9.9–3.0.7), showing identical results (unpublished data generated in the NaKo level III study). Surface calculation was validated using an in-house MathCad program confirming outcome of the body scanner software.

For each participant 140 measures were collected and stored in the LIFE research data base. These measures include 97 (linear) lengths and distances, 36 (curved) girths, 2 angles, weight, and the four aggregated characteristics ‘body mass index’ (BMI [7]), ‘waist to hip circumference ratio’ (WHR [24]), ‘waist circumference to height ratio’ (WHtR [1]) and ‘a body shape index’ (ABSI [3]).

### Preprocessing

**Fig 1:**
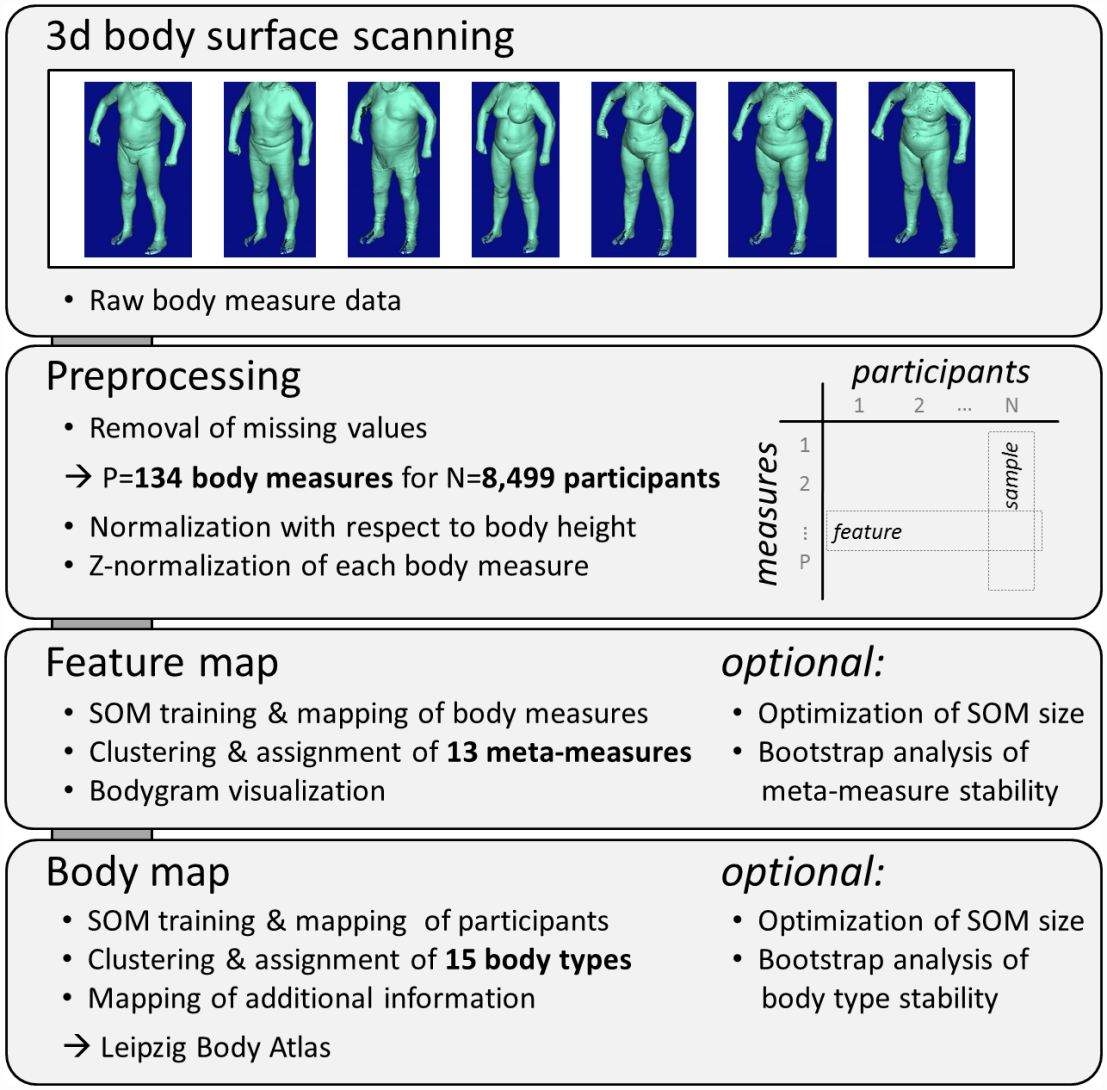
Workflow of SOM-based analysis of 3D body scanner data.

The P = 140 body measures of N = 9,892 participant constitute the P x N matrix of raw data (see Fig 1). Their preprocessing includes three steps: i) removal of missing values; ii) normalization with respect to body height; and iii) Z-normalization of each body measure:

1. The matrix contained 17,721 (≙ 1.4%) missing values for 1,868 participants which could not be estimated by the scanner software. In a first step, 46 participants were removed which show missing values for more than 50% of the body measures. In the second step, 6 body measures were removed which were missed in more than 5% of the participants (for a list see S1 Table). In the last step, the remaining 1,347 participants (≙ 13.6%), which still have missing values, were removed. The resulting data matrix without missing values comprises P = 134 body measures of N = 8,499 participants.
2. The body measures of each participant were then divided by the body height. This normalization step adjusts measures for body height and assumes that body shape linearly scales with body height.
3. Finally, each measure was Z-normalized, i.e. centralized with respect to its mean value averaged over all participants and divided by its standard deviation. Z-normalization makes the different measures comparable by providing a common scale in units of the standard deviation of each measure in the cohort.

### SOM clustering of body measures—‘feature map’ and bodygrams

We applied self-organizing map (SOM) machine learning for both, clustering of the 134 body measures into 13 meta-measures as well as for clustering of participants into body types using these meta-measures. SOM clustering into body types is described in the next subsection.

SOM machine learning projects the multidimensional features landscape, given by the body measures, into a two-dimensional map space [25]. We developed a comprehensive analysis workflow based on the SOM method. This so called ‘high-dimensional data portraying’ was previously applied to different data types in molecular proteome, genome and, first of all, transcriptome studies [26–30]. We applied SOM learning to the preprocessed body scanner data using standard parameter settings of the R-package ‘som’ [31]. A SOM size of 50 x 50 units was chosen after thorough adjustment of map resolution to optimally resolve the cluster structure inherent in the data (see S1 File). A larger SOM size does not change clustering results. Clusters of body measures were determined in this so called ‘feature map’ based on the patterns of the distance map which visualizes the mean Euclidean distance of each SOM unit to its adjacent neighbors (for details see [32]). A cluster is then defined as an area surrounded by units of (local) maximum distances (see S1 File). This SOM-based clustering outperforms alternative approaches such as principal component analysis and hierarchical clustering when applied to body scanner data as shown in S1 File. In total we detected 13 clusters of body measures termed ‘meta-measures’. These meta-measures collect between 2 and 27 single measures (see S1 Table). Their values were calculated as mean values averaged over the respective single measures in each of the clusters. Eleven singleton measures are not included in the clusters and excluded from downstream analyses. The resulting set of thirteen meta-measures anthropometrically characterizes each participant of the study. We Z-normalized the meta-measures of each participant to remove additive ‘offsets’.

### SOM clustering of participants—‘body map’

The second SOM called ‘body map’ was generated to cluster participants with similar body measures. For input data we utilized the thirteen Z-normalized meta-measures of all 8,499 participants of the study. We determined the minimum SOM size required to separate distinct body types by progressively increasing SOM size until the asymptotic range was reached and no new body types emerged (see S1 File). A size of 130 x 130 units was finally chosen. Each unit in the body type map collects one or more participants with similar body shape, or it remains empty otherwise. Body types were defined by separated clusters in the body map which contain at least 85 participants (≙1% of the cohort) to focus on robust and relevant clusters.

A visual description of body types and their mutual relations is achieved by staining the body map according to mean characteristics of the participants in each of the tiles such as age, gender, BMI, WHtR, WHR, and ABSI.

### Availability of data

Preprocessed meta-measures of all participants used in this study together with the assignment of gender, age, BMI and body type are provided as S2 Table. Raw data can be requested from the LIFE Consortium (www.life.uni-leipzig.de/en/).

## Results

### SOM-based analysis workflow for body scanner data

We developed a comprehensive workflow to process body scanner data in large epidemiological cohorts (see methods section and Fig 1). In the first step, raw data of body measures were preprocessed which includes the removal of missing values, body height- and Z-normalizations. Height normalization is supposed to minimize body size-scaling effects to focus our analysis on the variety of body shapes. Z-normalization is applied to make the different measures comparable in terms of a common scale.

After preprocessing, our data set comprises 134 body measures collected within the adult cohort of the LIFE study from 8,499 participants. We additionally included four classical body indices into the data set, namely ‘body mass index’ (BMI [7]), ‘waist to hip circumference ratio’ (WHR [24]), ‘waist circumference to height ratio’ (WHtR [1]) and ‘a body shape index’ (ABSI [3]). In the next steps we applied SOM machine learning either to cluster the body measures or the participants, respectively. Details are described below and in the methods section.

### 134 body scanner measures aggregate into 13 meta-measures

**Fig 2:**
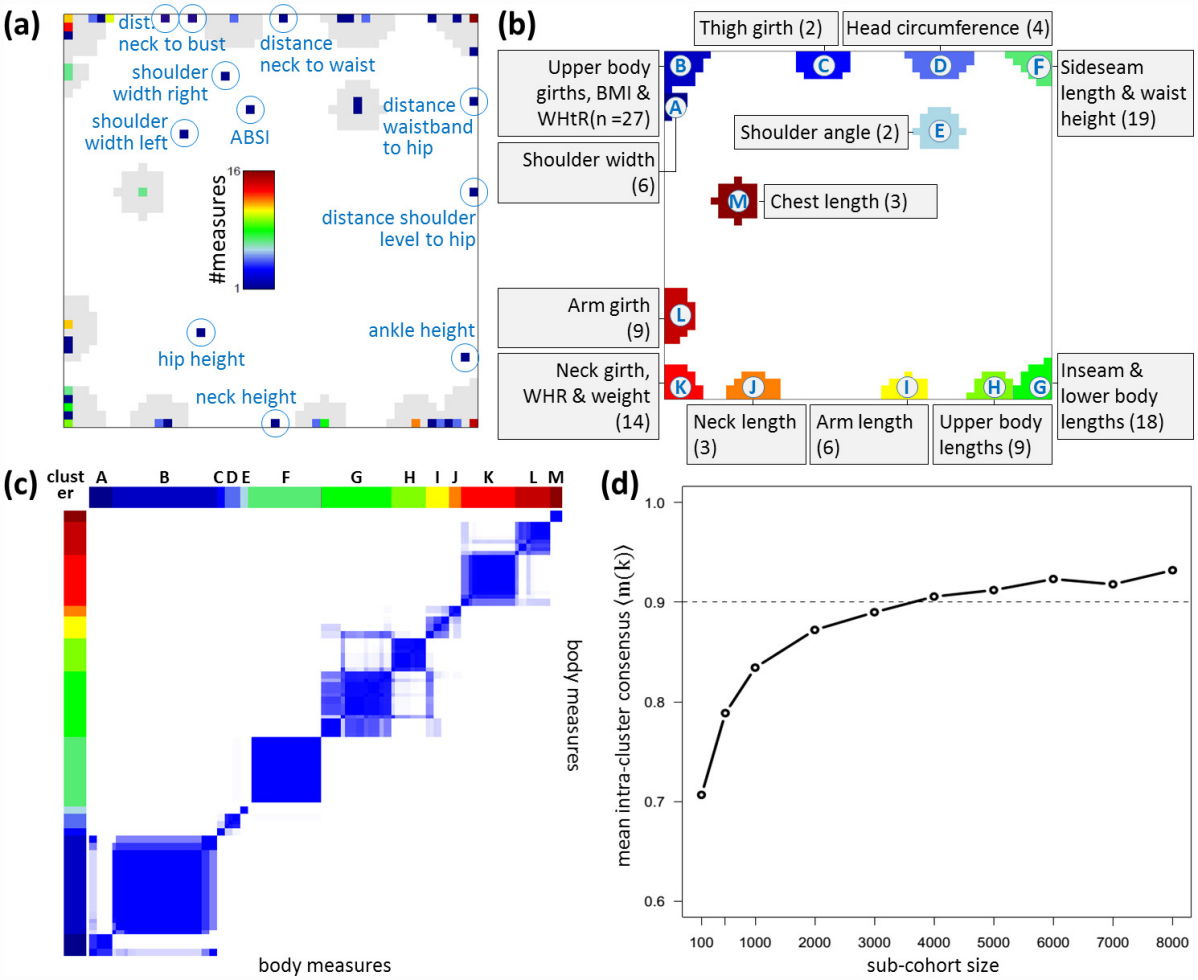
SOM cluster analysis of body measures. (a) The population map shows the localization of all 134 body measures in SOM space. The color code assigns the number of measures in each of the 50x50 SOM units, empty units are white. Singleton body measures not included in the meta-measure clusters are highlighted. (b) The same map as in panel (a), where thirteen clusters were detected and assigned with a ‘nick name’ characterizing the measures in the cluster. Their number per cluster is given in parenthesis. (c) Consensus cluster map of the features. Light to deep blue coloring indicates the increasing frequency of pairwise appearance of different features in the same clusters as determined in 100-fold bootstrapped SOM training and clustering. (d) Mean intra-cluster consensus as a function of the number of participants used in consensus clustering.

A feature map of preprocessed body scanner data is generated using SOM machine learning [25]. A grid size of 50 x 50 was chosen to achieve stable clustering of the 134 body scanner measures into thirteen clusters (see S1 File). The SOM algorithm organizes continuous features, here body measures, within a two-dimensional grid such that features with similar profiles across the cohort locate at the same or at close positions, whereas features with dissimilar profiles are found in different regions of the map. Fig 2a shows the localization of each body measure in the feature map. Each dot represents at least one individual measure. If multiple measures are located at the same position, their number is indicated by the color scale given within the figure: blue dots represent positions with 1 single measure, dark red represents a position with 16 measures. Importantly, the distances between the body measures in SOM space are scaled non-linearly, i.e. they expand in regions highly populated with features and decrease in sparsely populated regions. We make use of this property for the unsupervised determination of clusters of features with similar profiles. We obtained thirteen clusters termed meta-measures which contain from 2 to 27 individual body measures (Fig 2b). The meta-measures were labeled with capital letters following clockwise order in the feature map. This way the meta-measures were sorted according to the mutual similarity of their profiles which only partly agrees with their anatomical assignment. Meta-measures in the top left region of the feature map mainly refer to girth measures, whereas those in the bottom right region to length measures. For example, cluster ‘I’, called meta-measure ‘arm length’, contains six related measures, namely ‘arm lengths left & right’, ‘arm lengths to neck left & right’, and ‘up arm lengths left & right’ (see S1 Table for the complete list of meta-measure assignments). The localization of length and girth measures in opposite areas of the map reflects anti-correlated behavior, i.e. larger girths on average associate with smaller lengths and vice versa. Another meta-measure ‘G’, which was termed ‘Inseam and lower body lengths’, is a cluster of 18 features mainly related to heights drawing on the lower body such as ‘sideseam lengths left & right’, ‘buttock height’, ‘knee height’ and ‘distance neck to knee’. Please remind that the meta-measures refer to scaled body measures in relation to body height.

We judged the stability of our cluster assignments with regard to variation of the cohort composition using consensus clustering on randomly sampled sub-cohorts of 4,000 participants (≈ 50% of the cohort) as described in S1 File. The consensus map reveals deep blue squares along the diagonal which indicate stable clusters of body measures which grouped together in most bootstrapping iterations (Fig 2c). Light blue off-diagonal regions indicate a residual level of uncertainty of cluster assignment, e.g. between clusters G and H. The consensus map indicates a clear and robust cluster structure inherent in the body scanner data. This is further supported by the mean inter-cluster consensus value of 〈*m*(*k*)〉 = 0.91, meaning that feature pairs from the same cluster (as classified using the full cohort) attain common cluster assignments in 91% of the bootstrapping iterations.

Finally we studied cluster stability as a function of cohort size using partial inter-cluster connectivity for consensus clustering of randomly sampled sub-cohorts of sizes ranging from 100 to 8,000 participants (Fig 2d). It turned out that the mean inter-cluster consensus value asymptotically levels off at about 0.9 for cohorts greater than 4,000, indicating robust clustering. For cohorts smaller than 1,000, clustering of features becomes rather unstable.

### Bodygrams

**Fig 3:**
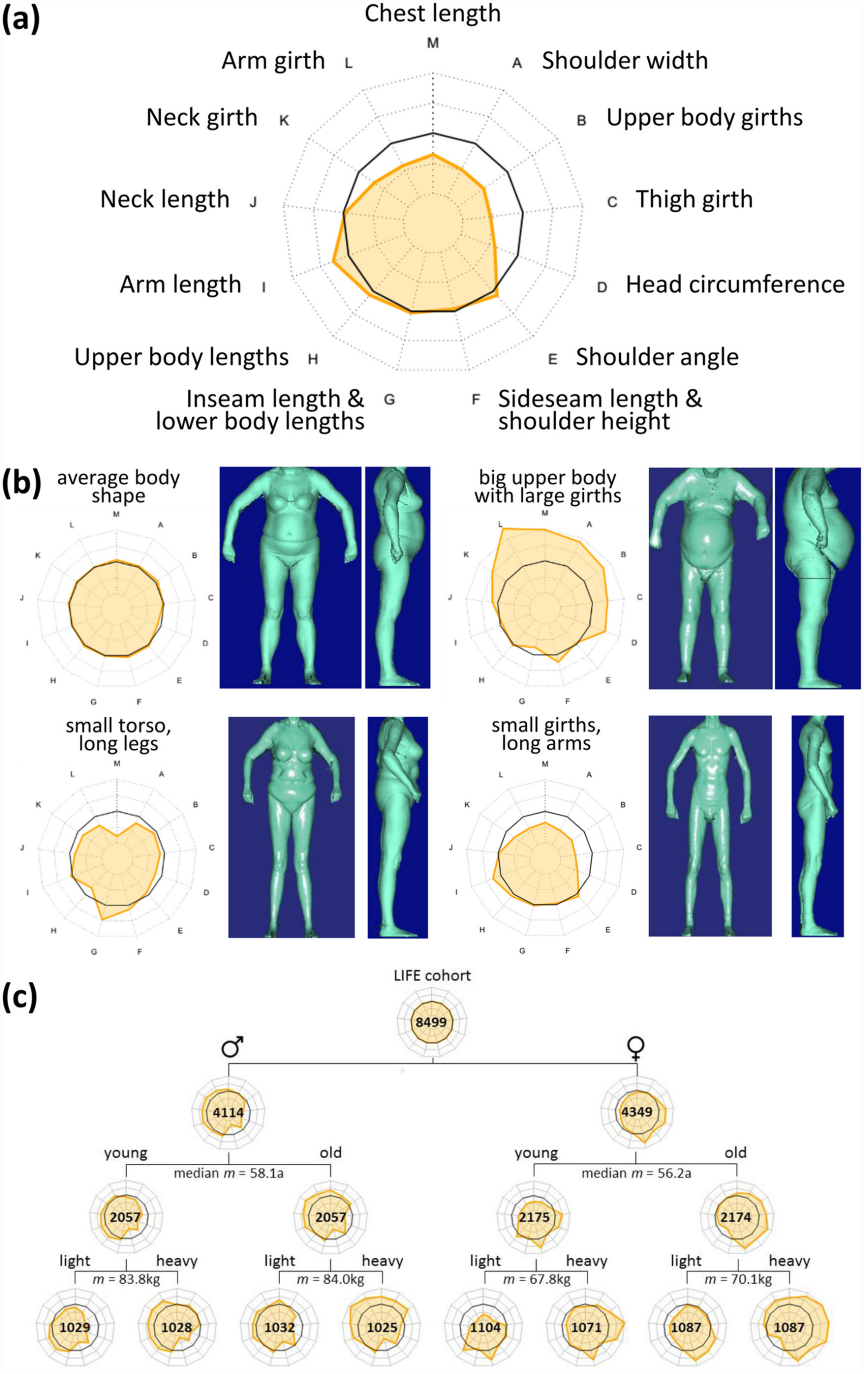
Visualization of body shapes using bodygrams. (a) Bodygrams are polar diagrams with the meta-measures in Z-units as axes. The black polygon refers to Z = 0. (b) Example bodygrams and the corresponding body surface scan images of selected participants. The dotted ‘cobweb’ lines indicate deviations from the mean in units of +/- 1, 2 etc. standard deviations. (c) Bodygrams averaged over all men (left branch) and women (right branch). The gender-specific groups were further stratified into young and old men/women and also light and heavy persons. The numbers indicate the size of the respective sub-cohort. Note that data was not complete for all participants.

We visualize the values of the thirteen meta-measures using a polar diagram representation called bodygram where the ordering of polar axes was chosen according to the ordering of meta-measures in the SOM (Fig 3a). The black polygon refers to Z = 0 and thus to the mean value of each measure averaged over the cohort. In the following the terms ‘big’, ‘small’, ‘long’ and ‘short’ relate to these average values.

Fig 3b shows four bodygrams of selected participants together with the corresponding body surface images. They reveal that relatively tall and thick people and also participants with short and long extremities can be easily identified by sections of the bodygram largely deviating from the Z = 0 baseline. The bodygram enables a simple shape-based perception of anthropometric phenotypes.

In the next step we generated mean bodygrams of sub-cohorts specified by gender, age and weight (Fig 3c). Comparison of the female and male bodygrams reveals typical gender-specific differences between body shapes: Men show higher values of torso and extremities lengths, whereas women show larger sideseam lengths and thigh girths. In general, these gender specifics are more pronounced for older people (especially women) whose upper body girths and shoulder angle markedly increase. Women also show larger (relative) ‘head circumference’ values than men. Larger body heights of men thus do not associate with a proportional increase of their head circumferences. We further stratified younger and older women and men with respect to their body weight: For young men a high weight associates with longer length measures and girths (upper body, thigh), whereas for older men a strong increase of upper body girth and shoulder width is observed. Higher weights are trivially found for obese as well as ‘strong’ men. Interestingly, younger overweight women show extraordinarily large thigh girths, whereas older women also show arm and neck girths beyond their average values.

This simple approach clearly shows that gender, age, weight and BMI reflect multiple characteristics of the body shape as seen by the meta-measures, whereas the former, virtually one-dimensional parameters are not able to comprehensively describe the multidimensional diversity of the body shape.

### The LIFE-ADULT cohort splits into distinct body types

**Fig 4:**
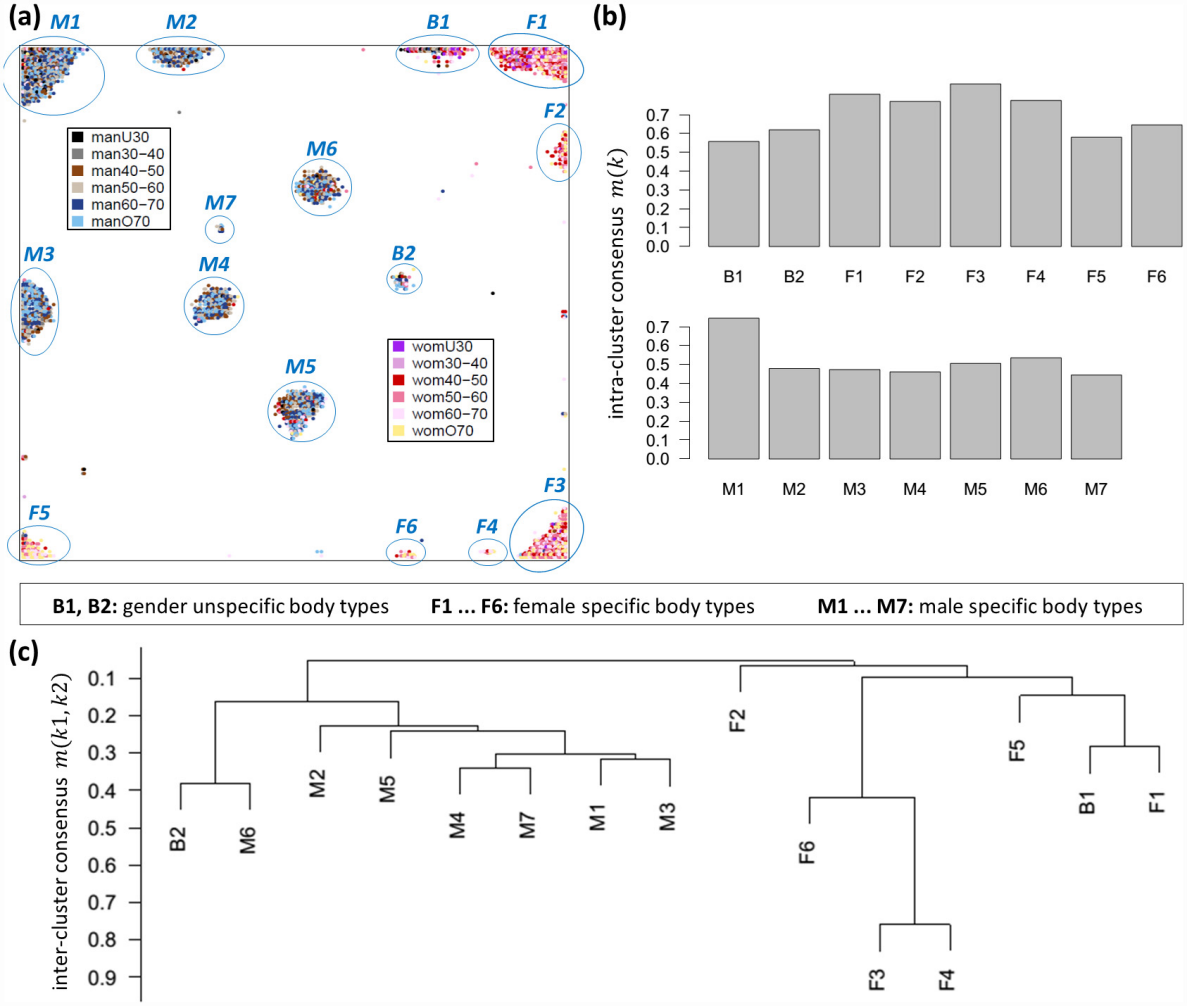
SOM cluster analysis of the participants. (a) The body map projects the 8,499 participants into a two-dimensional grid. It reveals 15 distinct groups of participants with similar body shapes defined by separated cluster regions. Each dot in the map represents one participant and is colored according to its gender and age stratification as given in the legend within the map. (b) Intra-cluster consensus values for each of the body type clusters reflect the degree of reliability and stability. (c) Hierarchical cluster dendrogram of body type clusters. Inter-cluster consensus values were used as similarity measure to define the branching heights of the dendrogram.

After clustering of body measures we clustered the participants into body types using SOM machine learning. We trained SOMs of increasing sizes with the meta-measure data of the LIFE cohort. We found that the number of resolved body (type) clusters increases until a SOM size of 130 x 130, and then levels off at a number of fifteen clusters (see S1 File). Hence, a SOM size of 130 x 130 was required to achieve sufficient resolution of the clusters inherent in the LIFE cohort of nearly 8,500 adult participants. These clusters either contain mainly women (labelled as F1…F6), men (M1…M7) or participants of both genders (B1 & B2) (see Fig 4a). A few participants could not be assigned to any body type cluster (n = 96, ≙ 1.1% of the cohort). They were located in isolated units of the body map and will be addressed in follow-up analyses.

We generated a consensus matrix by applying 100-fold bootstrapped clustering using sub-cohorts of 8000 participants. The stability of body type clusters was then evaluated using intra- and inter-cluster consensus values: Body types with high intra-cluster consensus (F1-F4, M1) can be regarded as compact and stable, while clusters with lower values (B1, F5, M4, M7) are more uncertain (Fig 4b). Note that intra-cluster consensus and number of participants in the body types are correlated with Pearson correlation coefficient of r = 0.5 indicating a direct relation between the size of the clusters and their stability: Highly populated body types collecting prevalent body shapes tend to be more stable than rare ones.

Inter-cluster consensus estimates the degree of overlap between two clusters. We utilized these values to generate a dendrogram of body types using hierarchical clustering with single linkage (Fig 4c) which reveals a high degree of similarity between the types F3 and F4 located together on the outer branches of the dendrogram. Further body types stratify successively until two major, gender-specific branches remain: the leftmost branch contains mainly men (M types and B2), the rightmost one mainly women (F types and B1).

### Resolution of body types depends on cohort size

**Fig 5:**
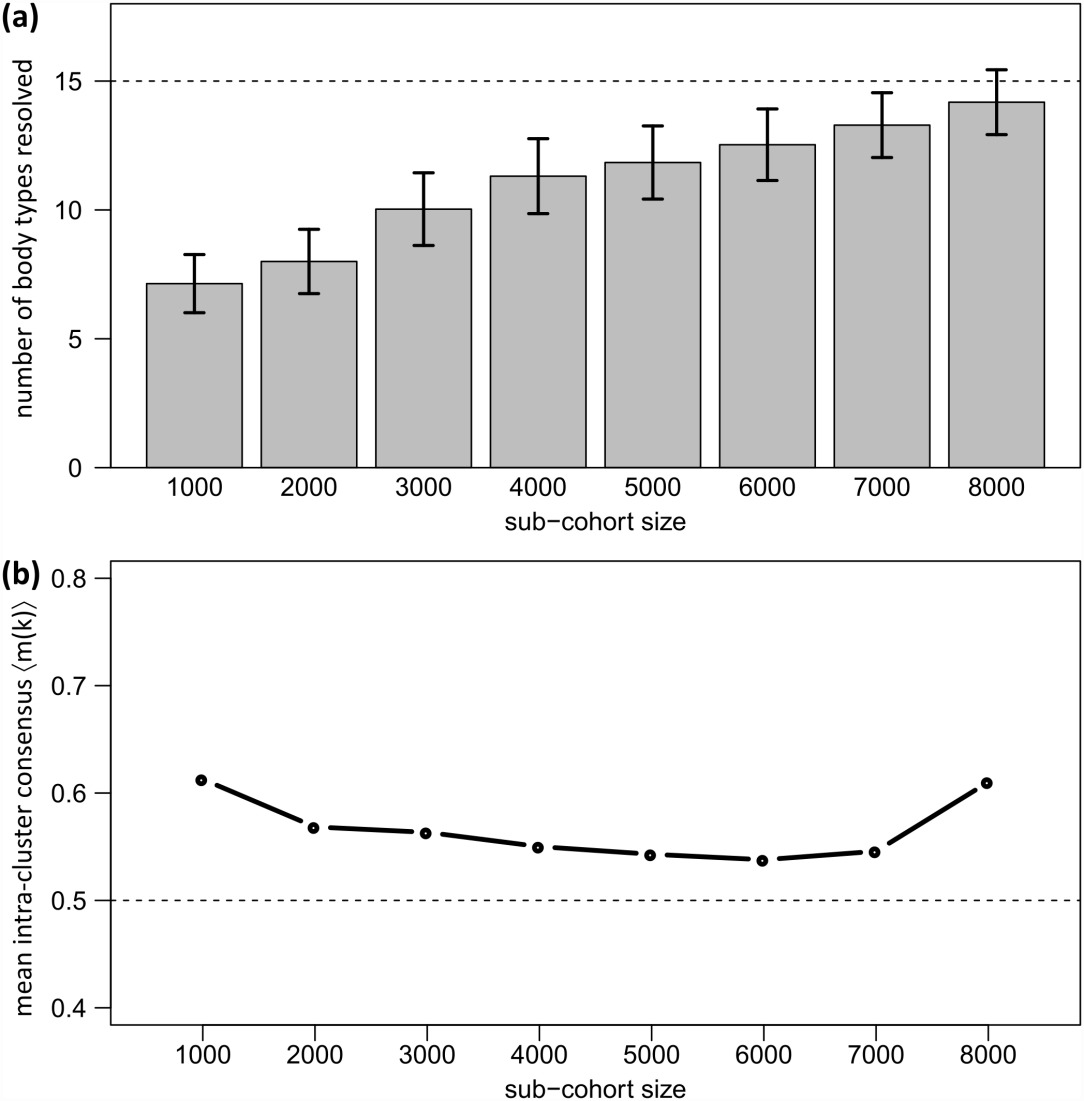
Number of body type clusters resolvable and their stability as a function of the cohort size. (a) Cluster numbers and corresponding standard deviation are obtained using 100-fold bootstrapped clustering for different sub-cohort sizes. (b) Mean intra-cluster consensus is given as robustness estimate for each sub-cohort size.

Stability analyses suggest that resolution of body types and the ability to detect rare body types increases with the number of participants in the cohort. Here the question arises how many clusters are generally detectable given a certain cohort size. We applied bootstrapped clustering using different sub-cohort sizes ranging from 1,000 to 8,000. We then counted the number of clusters in each of the bootstrapping iterations and for each of the different sub-cohort sizes to estimate the cluster number and their robustness as a function of the cohort size. The number of detectable body types increases with cohort size, as expected (Fig 5a). Moreover, the increase of the mean number of body types as a function of sample size does not seem to flatten, suggesting that the number of body types detected will exceed 15 in larger cohorts. The variability of cluster numbers is calculated as standard deviation within the 100 bootstrapping iterations for each sub-cohort size. It slightly decreases with increasing cohort size (Fig 5a), while the stability (i.e. mean intra-cluster consensus values) increases (Fig 5b) indicating that clusters become more stable and their number possibly settles in a sufficiently large cohort exceeding sample size of LIFE_ADULT with 10,000 participants. In particular, the maximum stability level of 〈*m*(*k*)〉 > 0.60 was achieved only for cohorts larger than 8,000. Smaller sub-cohorts with less than 6,000 participants give rise to a decrease to a value of about 0.50, accompanied by a decrease of the cluster number to about 12. This leads to the important conclusion that cohort size to a large degree determines the number of detectable body types. This result is expected and implies that the detection of ‘rare’ body types in terms of stable clusters requires a sufficiently large cohort size. The fifteen clusters detected in the LIFE cohort thus refer to the cohort size used and might potentially be further refined in larger cohorts in the future.

### The LIFE body types

**Fig 6:**
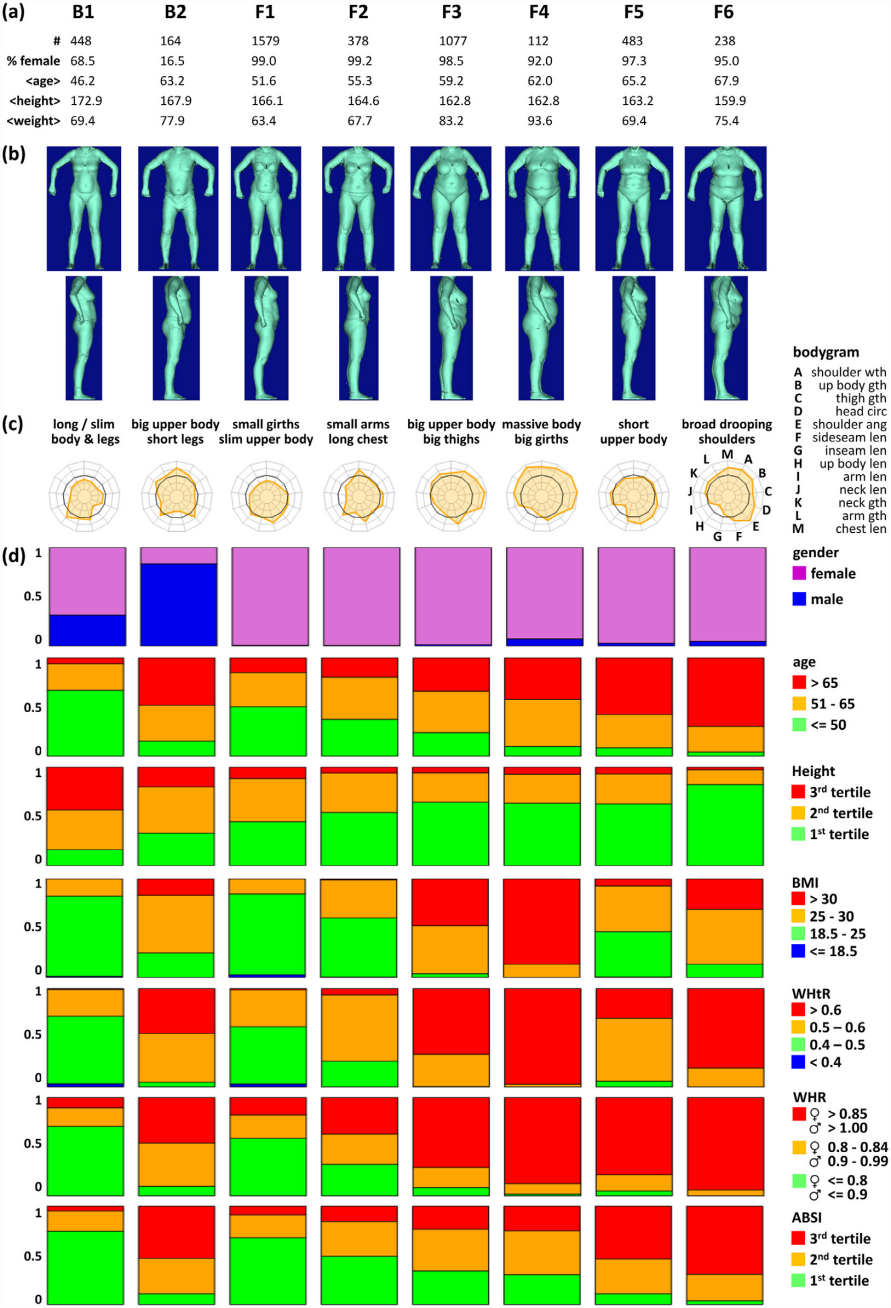
Characteristics of the B* and F* body types.

**Fig 7:**
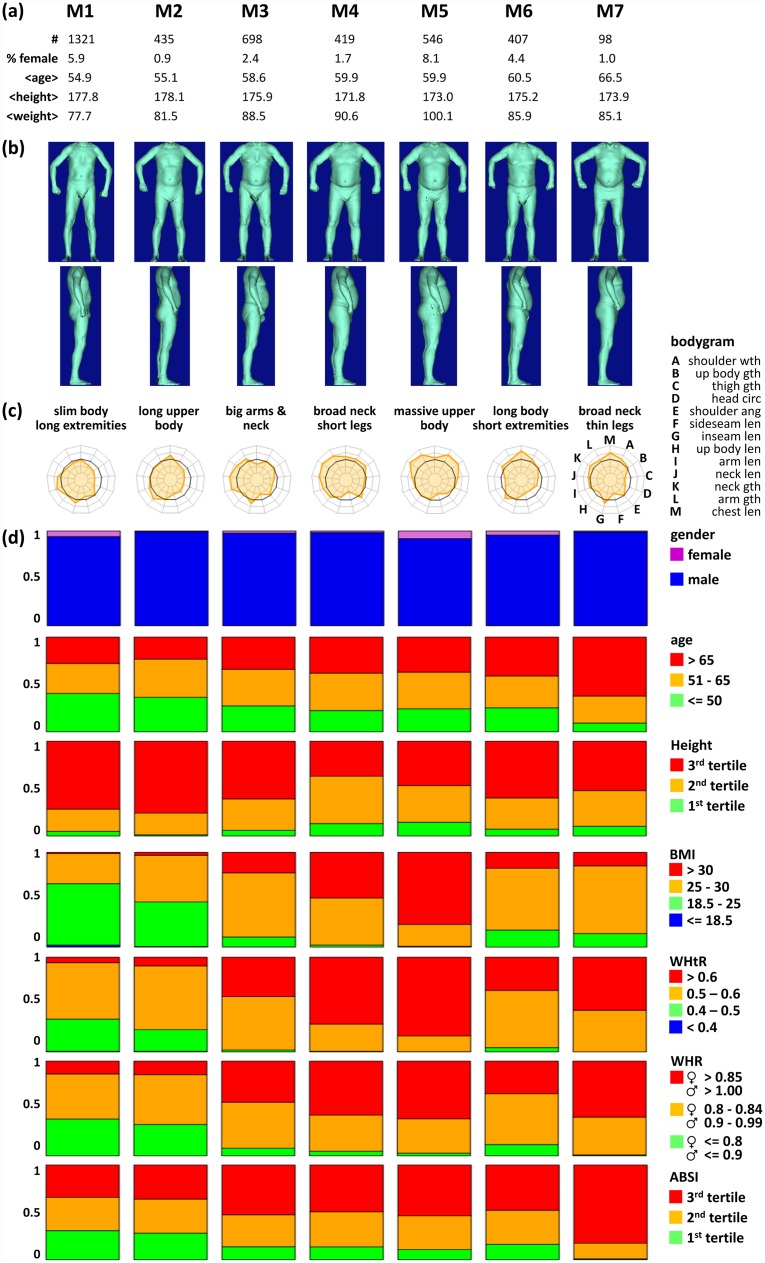
Characteristics of the M* body types.

Figs 6 and 7 characterize the 15 body types which were arranged according to gender composition: Body types B1 and B2 comprise larger fractions of both male and female participants, F1 to F6 mainly women, and M1 to M7 mainly men (see stacked bar plots in Figs 6d and 7d). Each body type typically comprises a few hundreds of participants covering the range between about 1% and 18% of total cohort size. Our clustering thus considers both rare and frequent body types. The mean age per cluster ranges from 46.2 to 67.9 years (Figs 6a and 7a). Please note, that participants younger than 40 years are strongly under-represented within LIFE-ADULT (5.5% in total).

The body surface images reflect a broad diversity of body shapes behind the clusters which include long and lean (B1, M1 & M2) and short and thick (F4 & M4) persons, but also short participants with short or long legs (M4 / F5) and arms (F2 / M3), respectively (Figs 6b and 7b).

Bodygrams visualize mean meta-measures of each body type (Figs 6c and 7c). They reveal that each body type features a unique combination of meta-measure characteristics. For example, both M6 and M7 are characterized by long upper bodies, but they differ in the size of their limbs. Hence, bodygrams together with body surface images provide a thorough characterization of body types emphasizing complementary aspects of body shape.

We further use barplots to characterize each body type with regard to gender, age and body height, and also the indices BMI, WHtR, WHR, and ABSI (Figs 6d and 7d): Gender composition facilitates the classification into male, female, and mixed body types. Age distribution of body types reveals almost balanced compositions (most notably F3 and M1), but also body types enriched with young (B1, F1 & M1) and old participants (F5, F6 & M7). Height distribution reveals enrichment of smaller participants in female, and taller participants in male body types as expected, with some body types collecting particularly small (F6; older women) and tall participants (M1, M2; younger men), respectively. Our results clearly reveal gender specifics in terms of size and shape of the human body subsumed as allometric differences between the sexes for most of the body types [33].

The BMI and WHtR characteristics of the body types resemble each other. In particular, obese participants (BMI>30) mostly show ‘apple-type’ body shapes (WHtR>0.6) whereas normal weight participants (18.5<BMI<25) are mostly ‘pear’-shaped (0.4<WHtR<0.5) [34]. WHR strongly resembles the WHtR patterns, but it tends to assign more participants to obese state. In general, obesity as seen by BMI, WHtR and WHR indices is distributed over diverse body types. ABSI is a body shape index reported to be non-correlated to other body measures and indices [3]. The barplots in Figs 6d and 7d yet reflect the trivial tendency of older participants (in body types F5, F6 & M7) to show higher ABSI, i.e. higher mortality hazard. WHR and WHtR show a similar age dependency, which in turn leads to an indirect association of those indices and ABSI.

In summary, the body types reveal very heterogeneous body shapes with specific characteristics beyond that of traditional body indices. In general, we found eight body types which virtually exclusively comprise pre-obese and obese participants (>80% participants with BMI > 25; body types F3, F4, F6, M3-M7). This fine resolution of participants with high BMI, WHtR, and WHR shows that obesity associates with different body shapes and fat distributions, e.g. mainly at the torso (M5), arms (M4), or thighs (F3 & F4). This important result potentially has impact on profiling obesity and obesity related health hazards.

### The Leipzig Body Atlas

**Fig 8:**
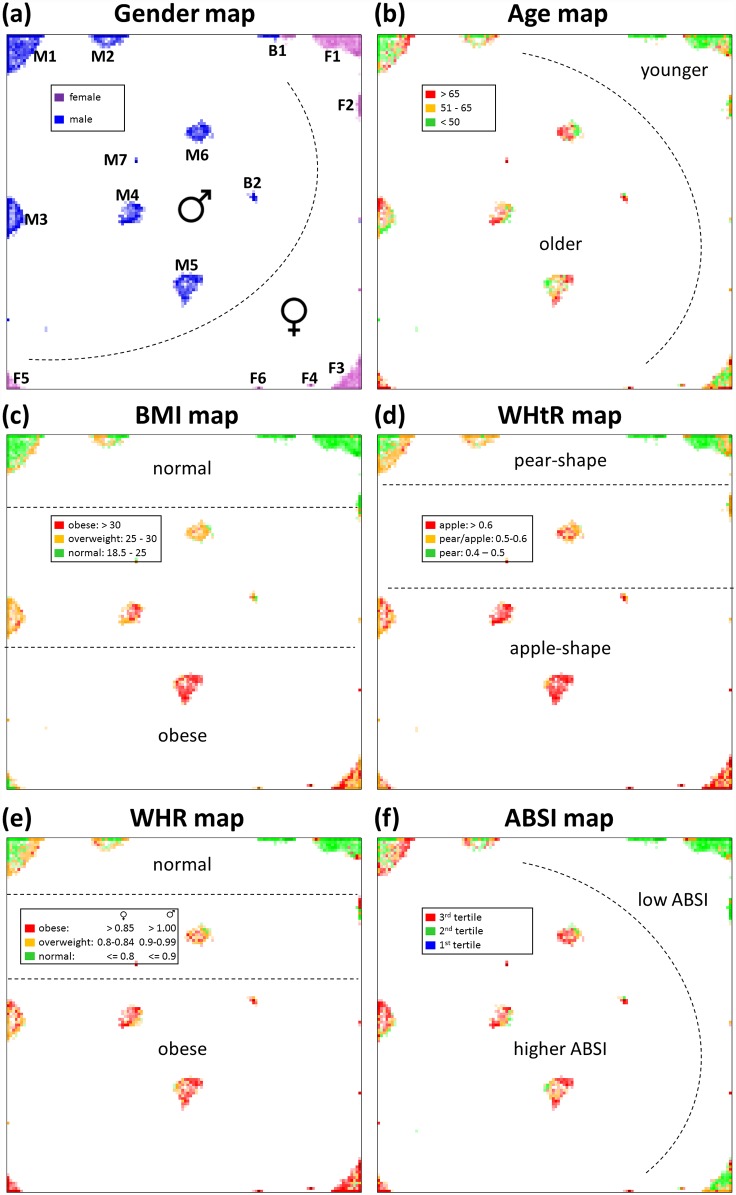
Towards the Leipzig Body Atlas. Body maps color coded for (a) gender, (b) age, (c) BMI, (d) WHtR–fruit types, (e) WHR, and (f) ABSI mortality index. Each colored pixel refers to the mean value averaged over all participants mapped to a particular SOM node.

To describe mutual relations between body types we visualize the clusters along with associated characteristics in the body map (Fig 8). In general, one finds a systematic shift of these characteristics along different directions in the map as a result of their incremental changes along the different body types. For example, gender changes predominantly in diagonal direction where women are located mostly in the right and bottom part, and men mostly in the top left part of the map (Fig 8a). Mixed gender clusters were found in an intermediate area in-between the female- and male regions of the map. We characterized these mixed gender body types separately for men and women (see S1 File for details). The gender-specific bodygrams of men and women are very similar confirming the agreement of their body types although total body height and weight differ markedly between women and men also in the mixed clusters.

The age map reveals body type clusters of especially young (F1 & M1) and old (F5, F6 & M7) participants along the top border and in central position of the map, respectively (Fig 8b), but mostly clusters of participants with a broad range of age. In general, mean age of participants changes predominantly in horizontal direction where younger participants accumulate in the top left part of the map.

We also mapped the body indices BMI, WHtR, WHR, and ABSI into the body map (Fig 8c–8f): The BMI, WHtR and WHR maps reveal close similarity (but not identity) and a virtually one-dimensional distribution of increasing values from the top to the bottom edge of the map. The ABSI index shifts more in diagonal direction. It was reported to associate with mortality hazard [3] and is trivially associated with age (compare age and ABSI maps). We also generated tertile maps for each of the twelve meta-measures (S1 File). They visualize modular combinatorics of meta-measures in the body types with high multivariate granularity and complementary to the bodygrams.

## Discussion

We analyzed 3D body scanner data of a cohort of 8,499 adult participants sampled from the population of Leipzig which constitutes one of the largest data sets of 3D body scanner data currently available. We identified 13 meta-measures and 15 distinct body shapes which goes far beyond the three clusters of identified before [21]. We were able to show that these features are robust given the sample size available. The identification of robust meta-measures and body type assignment was an essential first step. From here we can proceed to investigate the associations of the 3D-body shapes with other phenotypic or genotypic traits, disease prevalences and health hazards. This is now becoming possible and will be reported based on the LIFE-ADULT data elsewhere. One obvious step is to investigate how the 3D-body shapes is linked to unhealthy body fat distribution. We have a subset of over 1,000 participants in whom abdominal visceral fat is quantified from MRI-scans. We also have extensive serum laboratory traits on energy and lipid metabolism and cardiovascular assessments [23].

Our study has several limitations. The LIFE-ADULT cohort contained equal proportions of women and men in the age of 40 to 80 years [23]. Only few data of younger participants were available in this cohort. In addition, the study population is a population with a typical middle European ethnicity. African, Hispanic, Asian and other ethnicities contribute to less than 1% each. To broaden the database we are presently initiating a collaborative project with the German National Cohort to obtain 3D-body scans in an additional large series of individuals aged 20–70 years.

We developed an analysis pipeline for 3D body scanning data. The preprocessing step comprises removal of missing values, normalization with respect to body height, and Z-normalization of the individual body measures. Z-normalization translates the body measures into a common scale, which is essential for the SOM algorithm that uses Euclidean distances as similarity criterion. Normalization of participants’ measures to their body height assumes a proportionality model that scales body measures linearly with body height. The body types defined in this publication consequently refer to human bodies artificially scaled to a common height. This scaling allows to distinguish body shapes rather than separating participants on an absolute height scale.

After preprocessing, the body measures provided by the scanner software aggregate into a roughly tenfold reduced set of meta-measures each representing a cluster of correlated single measures. Redundant information in the original data is thus mostly removed and dimensionality is reduced to relevant measures. In result, each participant is characterized by a set of meta-measures, each of which estimates one relevant dimension of body shape. In a second clustering step, participants were aggregated into fifteen body types. It can be expected that with larger sample sizes one may be able to detect more subgroups.

We provide a novel methodological option to stratify large epidemiological cohorts into a series of well-defined body types. The capabilities of SOM machine learning to accomplish body typing have been demonstrated, where particular strengths of this method can be seen in the visualization capabilities, multidimensional mapping, and non-linear scaling of the data. The resolution of our body types goes beyond a few main variables and reaches a high degree of multivariate granularity.

We also demonstrated that 3D-body typing needs sample sizes of several thousands of participants to obtain robust body types. The questions whether our body typing can provide new standards for anthropometric health classifiers and whether the concept of multidimensional ‘body types’ has advantages compared to, e.g., one-dimensional body indices requires further investigation. Forthcoming studies including tens of thousands of participants and, most importantly, association of the body types with health states, environmental conditions and lifestyle factors will allow judging the relevance of anthropometrical body screening in healthcare research. We expect that this work will open some of these perspectives.

## Supporting Information

S1 File Supplementary text. (PDF)

S1 Table Lists of body measures. Information about measures excluded from and included in the feature map, and assignment to the meta-measures.
(XLSX)

S2 Table Processed data table. It contains age, gender, BMI, body type and meta-measures of all 8,499 participants.
(XLSX)

## Abbreviations

Body surface image: Image showing the 3d body surface scan
Body scanner measure: Body measure derived from 3d surface scan
Body measure: Single anthropometric measure
Normalized body measure: Body measure divided by the body height. We use the terms ‘body measure’ and ‘normalized body measure’ as synonyms in the paper
Body scanner data: Data set comprising all body measures obtained by means of the 3d body scanner
Feature map: Self-organizing map of body measures
Meta-measure: Representative measure characterizing a cluster of body measures defined in the feature map
Body shape: Anthropometric proportions of the body. In our study it is defined by the set of meta-measures per participant
Body map: Second level self-organizing map that clusters participants according to similar body shapes
Body type: Mean body shape averaged over the body shapes in each cluster
Bodygram: polar diagram of the meta-measures characterizing the body shape or body type
Relevant dimensions: Independent coordinates required to characterize variability of body shapes
