# Supplementary material for: Novel Anthropometry Based on 3D-Bodyscans Applied to a Large Population Based Cohort
Source: PLoS One. 2016 Jul 28;11(7):e0159887. doi: 10.1371/journal.pone.0159887 (PMC4965021; doi:10.1371/journal.pone.0159887)
Supplement: S1 File — (PDF) [file pone.0159887.s001.pdf]

Novel anthropometry based on 3D-bodyscans applied to a large population based cohort

Henry Löffler-Wirth*, Edith Willscher, Peter Ahnert, Kerstin Wirkner, Christoph Engel, Markus Loeffler+, Hans Binder+

* corresponding author
\+ shared senior authorship

Supplementary text

1. Alternative clustering of body measures 2
2. Determination of the size of the feature map 3
3. Determination of the size of the body map 5
4. Consensus clustering to judge the stability of SOM clusters 6
5. Characterization of the mixed gender body types 7
6. Body typing using k-means 8
7. Leipzig body atlas – meta-measures 10
8. Frequency distributions of the age, body height and weight of the cohort and of the body types 12
9. Age-ranked profiles of the meta-measures of men and women 13
10. References 14

1. Alternative clustering of body measures

Several alternative approaches are available for data clustering. Here we applied principal component analysis (PCA) and hierarchical clustering (HC) to evaluate cluster structures in the body measure data. The plot of the first two components of PCA (Figure S 1a) and also that of higher components (not shown) does not reveal a clear cluster structure but rather a homogeneous distribution of data points. The HC dendrogram provides a branch structure, which however needs to be cut at a designated height to obtain distinct clusters (Figure S 1b). This choice of the threshold is crucial and represents a trade-off between too big and small clusters because the main branches split at different heights. Hence, proper definition of the height to cut the dendrogram into separated cluster branches remains unclear. Other clustering approaches, such as k-means, requires predefinition of the cluster number, which is unknown in most cases.

Note that PCA data space scales linearly with the mutual distances between the data whereas scaling of SOM space is weighted by local data densities. In consequence, SOM 'amplifies' local enrichment of data and provides a more pronounced cluster structure compared with PCA. In contrast, HC by definition splits the data into disjoint branches which in the particular case of body scanner measures provides no clear criterion for their separation. Contrary, SOM machine leaning of the body scanner data provides a clear cluster structure and cluster number (see below).

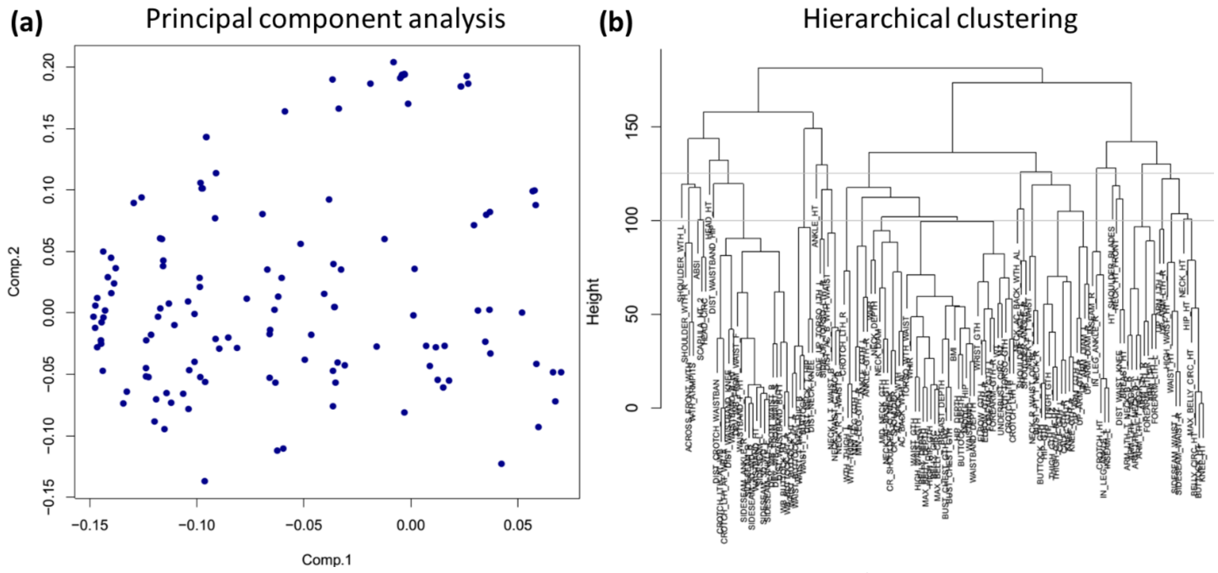

Figure S 1: Principal component analysis and hierarchical clustering of the body measures: (a) The scatter plot with the first two largest PCA-components reveals a virtually unstructured data cloud. Each dot represents one body measure. (b) Hierarchical clustering dendrogram of the body measures.

2. Determination of the size of the feature map

The minimum size of the SOM required to adequately project the body scanner data into a two-dimensional map space is an intrinsic property of the data. We have previously shown that the increase of SOM size beyond a certain threshold does not entail an increase of the number of data clusters [1]. We validate this finding for the body scanner data by training SOMs of increasing size using the same input data in each training run. The resulting cluster structure was inspected using so-called distance maps which clearly express the borders between different clusters in map space [2]. The distance map visualizes the mean distances between the map units and each of its four neighboring units. Particularly, our algorithm finds the SOM units referring to local maxima of their mean distance with respect to their neighbors. These pixels form 'halos' edging the relevant clusters in the respective distance map and enable robust determination of feature clusters in the SOM (see insertion in Figure S 2b). This SOM-based clustering outperforms alternative approaches such as principal component analysis and hierarchical clustering (see Figure S 1). The cluster structure of the feature map becomes clearer resolved with increasing number of units used (Figure S 2a). The number of resolved clusters first increases with the map size and then it converges to a number of thirteen clusters (Figure S 2b). Please note that this number refers to clusters only, which contain two or more body measures. We finally chose a map size of 50x50 units for establishing the body scanner feature map.

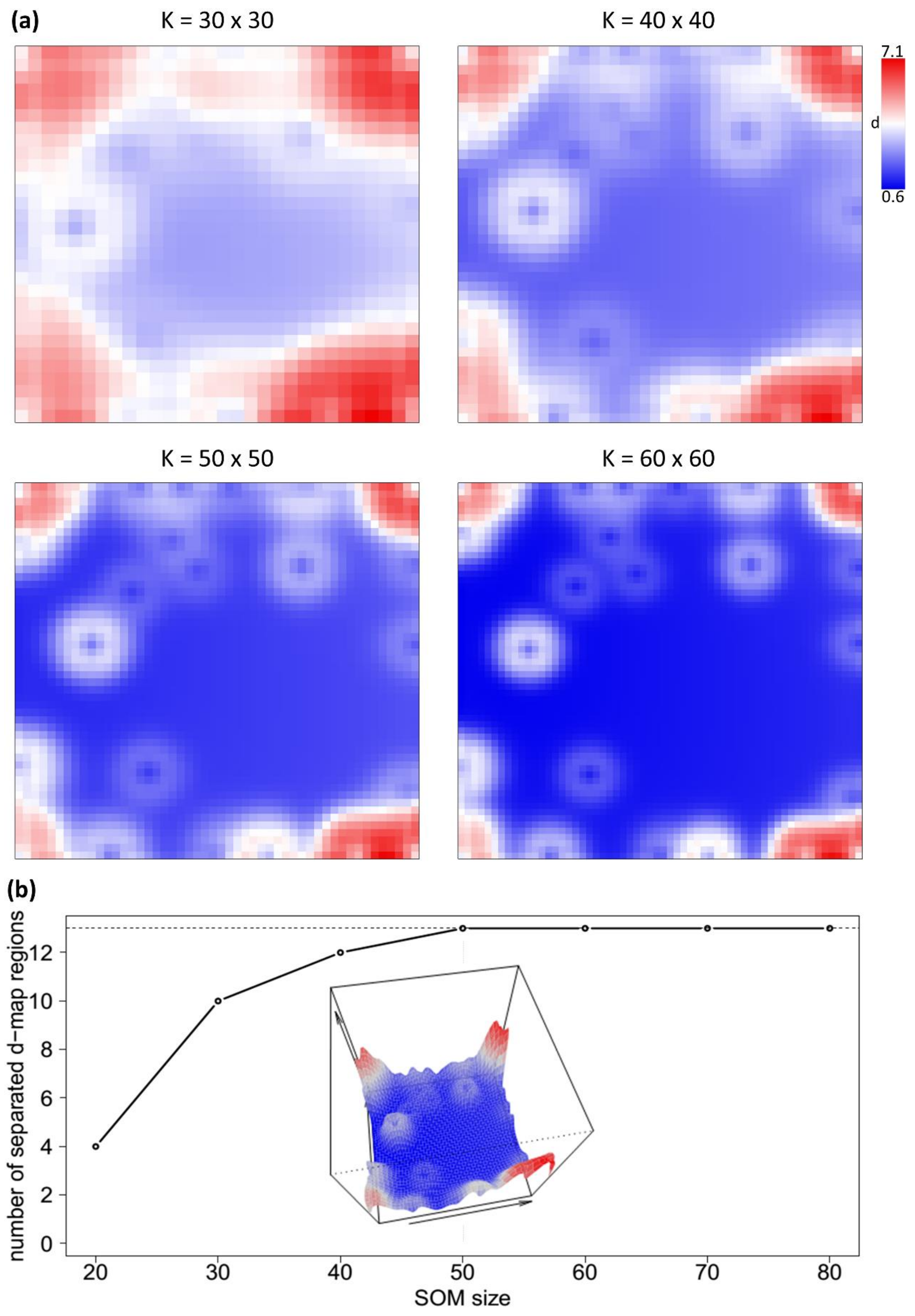

Figure S 2: Determination of clusters of body measures using the SOM distance map: (a) Distance maps of increasing size increasingly resolve the cluster structure inherent in the body measure data. Red and blue tiles indicate SOM units with large and small mean Euclidean distances to their adjacent neighbors. A cluster is typically characterized by a local minimum surrounded by a 'halo' of local maximum distances. (b) The number of cluster detected levels off for SOM sizes larger than 50 x 50. The distance map for the 50 x 50 SOM is shown as three dimensional 'perspective' image. It clearly indicates the 'volcano-crater' –like shapes of the clusters.

3. Determination of the size of the body map

We determined the minimum size of the body map by estimating the number of clusters as a function map size in analogy with our approach to estimate the size of the feature map. Granularity of the maps increases with size (Figure S 3a). The number of clusters levels off for a size greater than 130x130 (Figure S 3b). We therefore used this size for the Leipzig body type map.

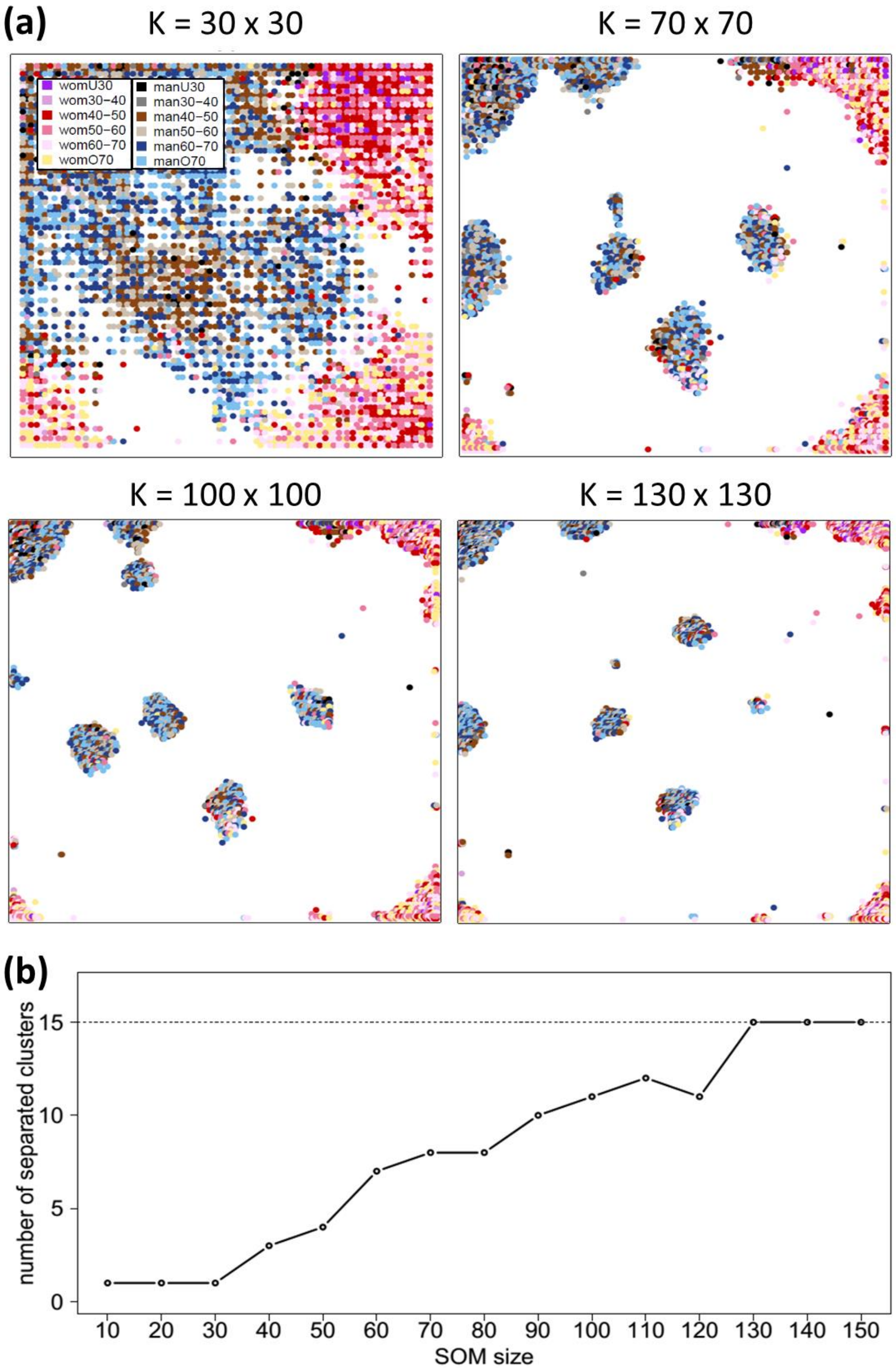

Figure S 3: Determination of clusters of body types using the SOM body map (a) Body type maps of increasing size reveal increasing resolution of the cluster structure. Each dot represents one SOM unit occupied by at minimum one participant. Note that up to nine participants are shown per body map unit. Coloring refers to age and gender (see legend). (b) The number of clusters detected levels off for SOM sizes larger than 130 x 130.

4. Consensus clustering to judge the stability of SOM clusters

We applied consensus clustering to assess meta-measures and body types provided by the feature map and body map SOM analyses, respectively. Consensus clustering is a bootstrapping method aiming at judging reliability of cluster assignments [3]. Bootstrapping uses sub-cohorts of a certain number of participants randomly sampled from the LIFE cohort to train SOMs and to extract clusters of body measures and types, respectively. It provides a quadratic consensus matrix M with the frequency of common cluster memberships for all pairwise combinations of body measures in a series of 100 resampling runs as elements. M is visualized as clustered heatmap collecting features frequently found within the same cluster into blue squares along the diagonal.

Consensus matrices for feature clusters were generated for random cohorts of sizes ranging from 1,000 to 8,000 participants. They were compared in terms of the intra-cluster consensus $m(k) = \langle \mathrm{M}_{i,j} \rangle_{i,j \epsilon k}$ and the inter-cluster consensus $m(k1, k2) = \langle \mathrm{M}_{i,j} \rangle_{i \epsilon k1, j \epsilon k2}$, representing the mean connectivity of all feature pairs of the same and of two differing clusters, respectively. The former measure estimates the stability of the individual clusters, the latter one estimates the degree of overlap between two clusters. Cluster assignments were obtained from the initial cluster analysis using all data.

The mean intra-cluster consensus $\langle m(k) \rangle$ finally estimates the overall degree of clustering stability given a particular consensus matrix and cluster assignments. A value of $\langle m(k) \rangle = 1$ indicates perfect clustering.

Consensus clustering was also applied to judge the stability of body type clusters upon systematic variation of sub-cohort size from 1,000 to 8,000 participants. For each size we performed 100 iterative SOM training runs with participants randomly sampled from the LIFE cohort. The mean intra-cluster consensus was then calculated based on the 15 body type clusters to assess their stability.

The maximum number of body type clusters distinguishable in a cohort of a given size was also determined. We utilized the consensus clustering results from the bootstrapping analysis and performed hierarchical clustering of the consensus maps to obtain a unified clustering for each of the sub-cohort sizes. The number of clusters was thereby determined by the mean number of resolved SOM clusters averaged over the respective 100 bootstrapping iterations.

5. Characterization of the mixed gender body types

Two of the body types contain a considerable fraction of both men and women. We characterized each of them separately for the men and women contained (Figure S 4). In general, respective bodygrams closely resemble each other however the mean body height and weights differ in gender specific fashion: male participants are, on the average, taller and heavier than female participants and vice versa, as expected.

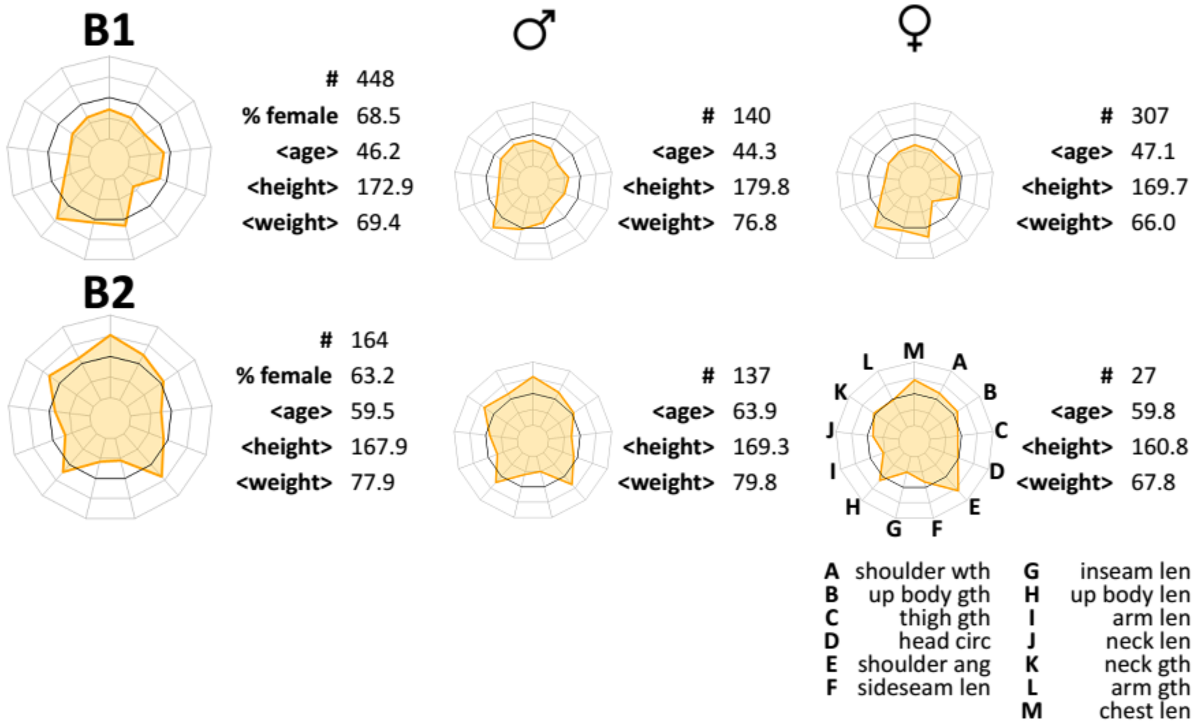

Figure S 4: Bodygrams and main characteristics of the mixed gender body types (left part) were separately stratified for men and women (right part). Note that the similar shapes of the body grams refer to different mean age, body height and weight of male and female cluster members.

6. Body typing using k-means

We applied k-means clustering as an alternative method to determine clusters of body types and to systematically study the relation between the number of clusters and their stability. The number of clusters k was increased from 2 to 10 and their stability was estimated using consensus clustering with 100-fold bootstrapping using 80% (= 6,799) of the participants [3]. Corresponding consensus maps visualize frequency of common cluster membership for all pairs of participants. The cumulative distribution function (CDF) aggregates consensus values into one curve. In theory, the area under CDF curves (AUC) is assumed to increase with k until the 'true' cluster number is reached [3]. Incremental change of AUC consequently serves as a measure to judge whether increasing the class number leads to a marked increase of clusters' stability or not. This ΔAUC progressively decreases with k indicating progressive amendment of cluster stability over the whole range of k studied where gain in stability however asymptotically vanishes for k > 4 (Figure S 5a).

Consensus map for k = 2 reveals a perfect clustering into two body types referring almost exactly to female and male participants, respectively. With increasing k one observes a gender effect of alternating cluster stability as seen by mean intra-cluster consensus (Figure S 5b). Particularly, clusters are uncertain for k = 3 whereas for k = 4 one again finds stable clusters where each two of them contain nearly exclusively men and women, respectively (Figure S 7). Further, the clustering algorithm decomposes the data into four distributions with mutually shifted means in this case (Figure S 6b).

The k-means algorithm obviously tends to generate clusters of similar size where for even k = 2 and 4 one gets relatively stable clusters of masculine or feminine body shapes whereas for k=3 and k>=5 the algorithm tends to generate a number of sub-clusters leading to a loss of overall cluster stability.

Finally we evaluated the effect of cohort size on cluster stability for k = 4 using consensus clustering and 100-fold bootstrapping. Mean intra-cluster consensus increases and exceeds 0.9 already for 2,000 participant (Figure S 6d).

In Figure S 7a we characterized the resulting four alternative body types more in detail. The two mainly male and two mainly female clusters distinguish younger and older participants with leaner and thicker body shapes, respectively. The four alternative body types distribute over the fifteen SOM clusters found in the main paper (Figure S 7b): a(lternative)M1 and aF2 further split into more granular types M1 to M3 for men and F1, F2 and F5 for women, respectively. Interestingly the mixed gender types (B1 and B2) contain mainly lean shape aF1 and thick body shape aM2, respectively.

In summary, k-means clustering provides similar gender-specific body shapes as SOM clustering. SOM clustering is however unsupervised with respect to k, the cluster number. Hence, the SOM method not only clusters data but it determines cluster number in contrast to k means which requires a preset number of clusters. Moreover, k-means clustering tends to divide normal distributed data into virtually 'equal' portions whereas SOM clustering enables identification of clusters with a wider range of population numbers due to non-linear scaling of the sampling space. Unsupervised estimation of k is crucial because the number of 'detectable' clusters increases with cohort size (see main manuscript).

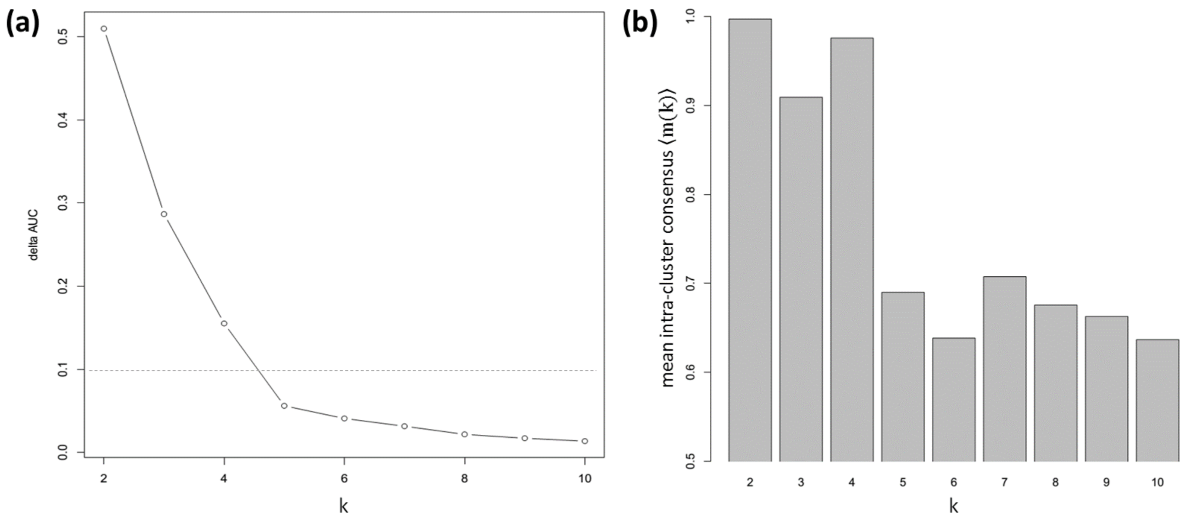

Figure S 5: (a) Increment of AUC of the corresponding consensus map CDFs (ΔAUC) and (b) mean intra-cluster consensus for increasing cluster number k.

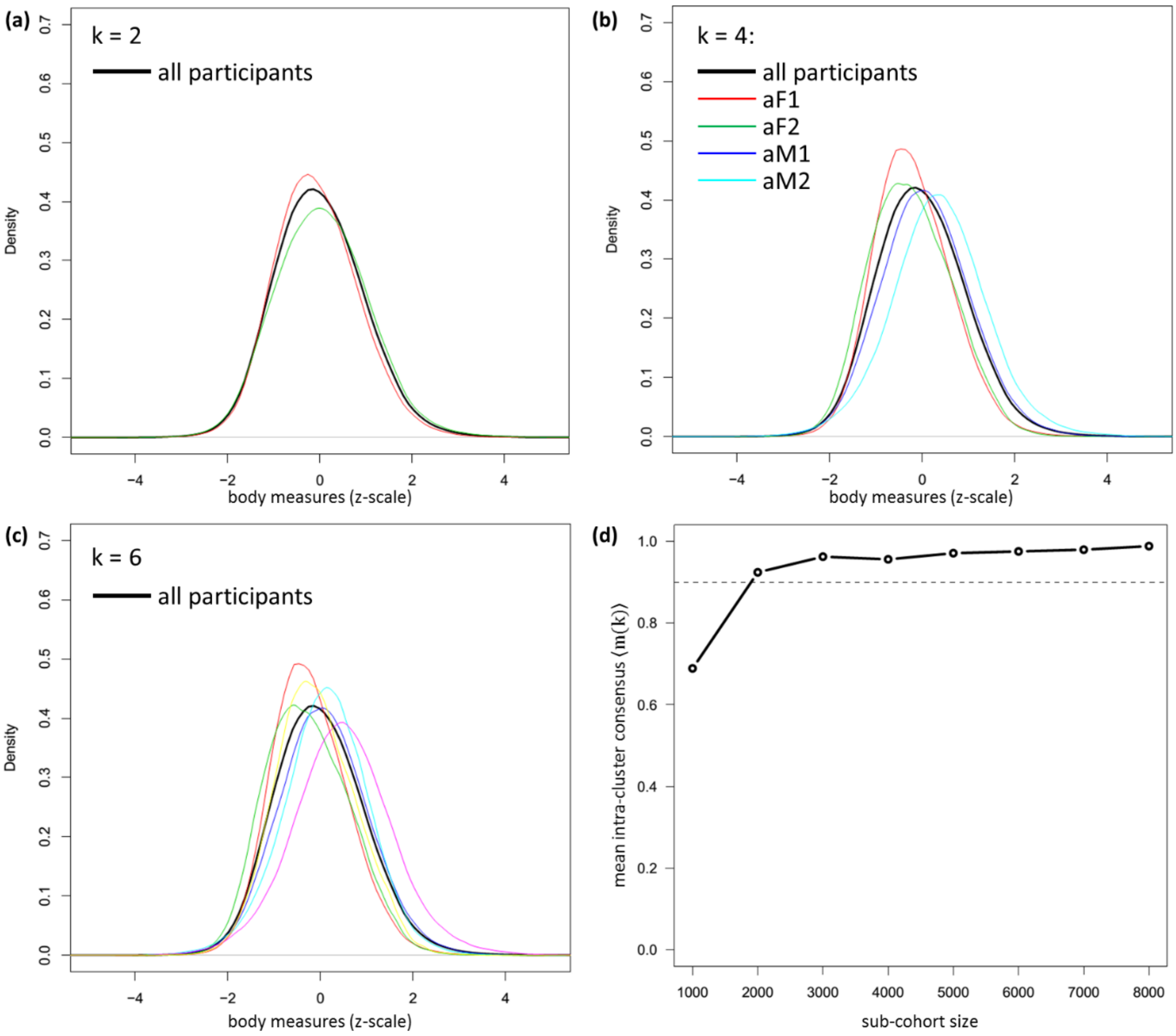

Figure S 6: (a-c) Data distribution for the resulting alternative body types using k = 2, 4 and 6. (d) Mean intra-cluster consensus is shown as a function of the cohort size using 100-fold bootstrapped consensus clustering of the sub-cohorts into k=4 clusters.

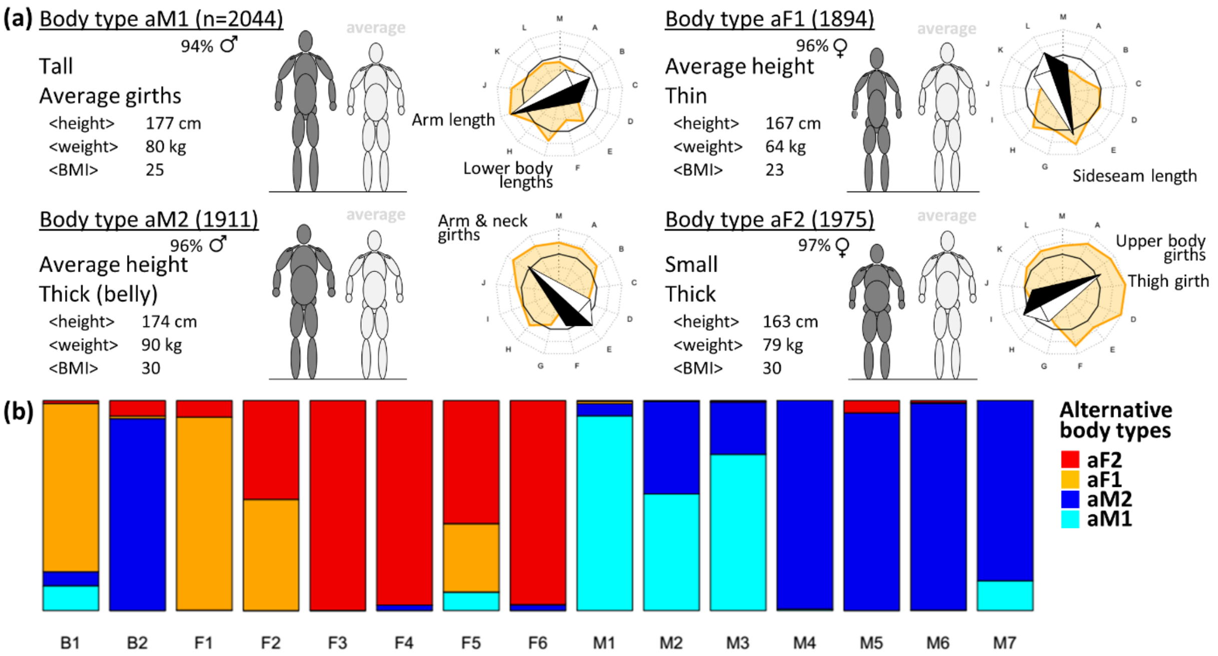

Figure S 7: (a) Characteristics of the four alternative body types. The ‘compass needle’ points into direction of large meta-measures exceeding the respective mean over the population. Note that they point roughly into four mutually perpendicular directions. (b) Distribution of the four alternative body types over the 17 body types as determined in the main manuscript using SOM clustering.

7. Leipzig body atlas – meta-measures

The body map projects the multidimensional distribution of body measures into a two-dimensional plot. In the main paper we colored the body map according to gender, age and different aggregated measures such as BMI, WtH and ABSI. Here we show body maps colored according to each of the meta-measures of the participants in each map unit. In general, most meta-measures match to the clusters and change systematically within the maps (Figure S 8). However, some meta-measures (e.g. ‘E’, and ‘M’) do not fit to the clusters and heterogeneously distribute between them. Interestingly, these measures form clearly separated sub-clusters that enable stratification of the cluster members with respect to selected measures.

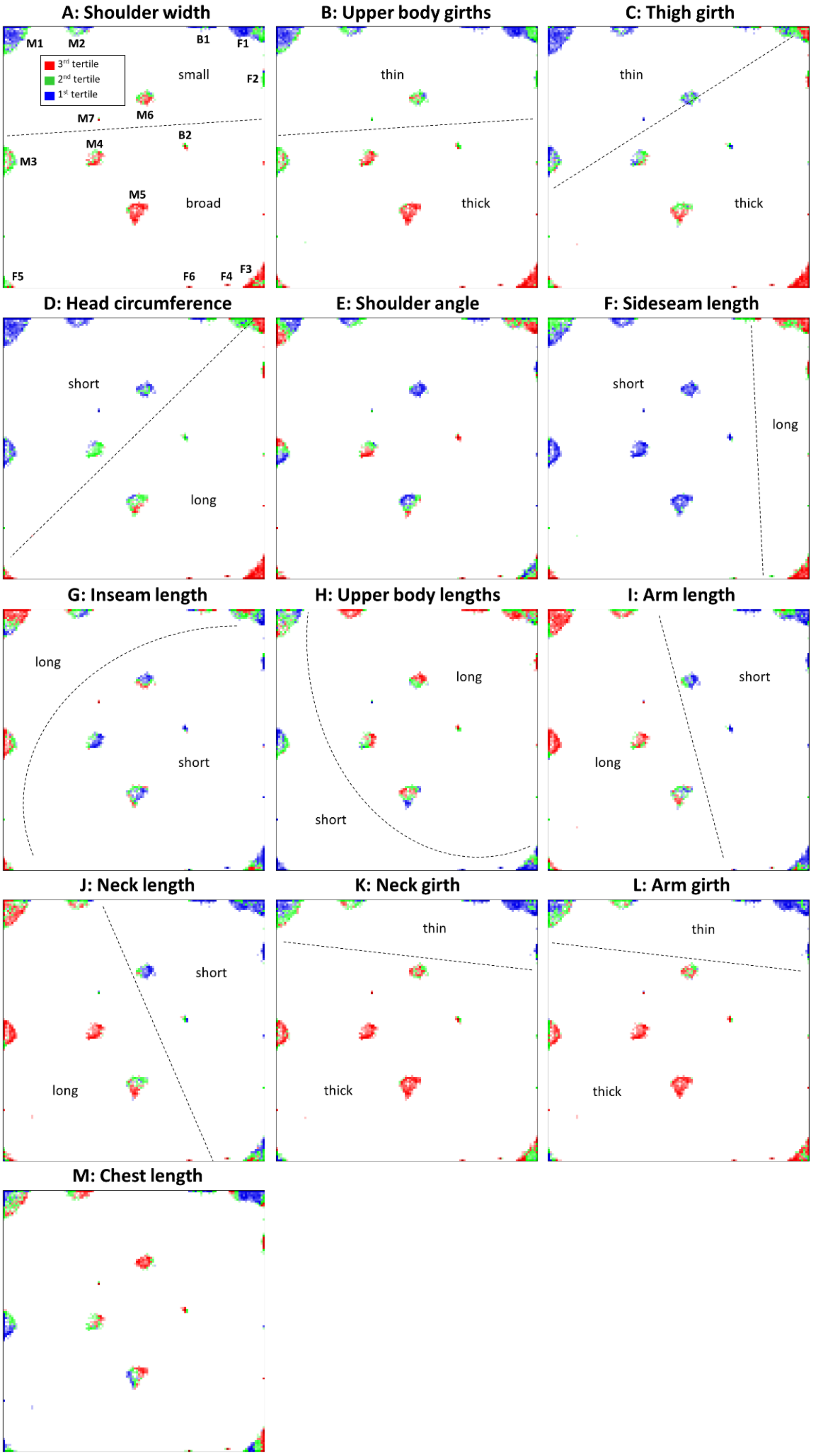

Figure S 8: Body maps color coded according to the tertiles of the thirteen meta-measures.

8. Frequency distributions of the age, body height and weight of the cohort and of the body types

We generated histograms to evaluate the frequency distributions of age, body height and weight in the LIFE cohort and in each of the body type clusters (Figure S 9). The overall age distribution reflects the sampling frequency of volunteers which is strongly enriched in participants older than 40 years. The frequency distributions of the body types reveal a shift from younger to older participants in the body types F1 to F6 and M1 to M7, respectively (Figure S 9a). The overall distribution of body height is Gaussian for men and women with mutually shifted maxima, as expected (Figure S 9b). Weight distribution is slightly asymmetric with a heavy tail towards higher weights for men and women, again with shifted maxima. Almost all partial height- and weight-distributions of the body types are single peaked and cover limited parts of the respective overall distributions.

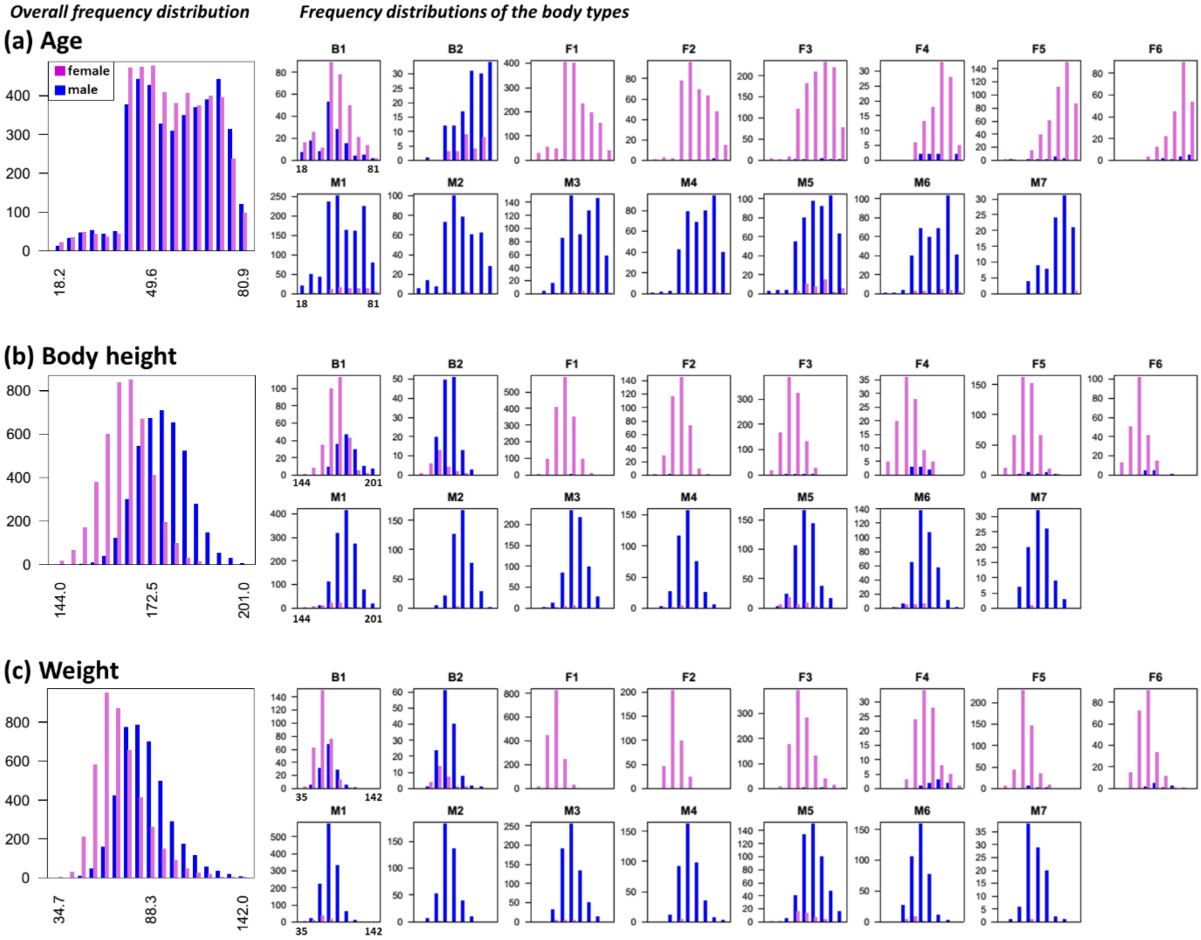

Figure S 9: Frequency distributions of (a) age, (b) body height and (c) weight of all participants in the cohort (left part) and of the participants stratified according to the 15 body types (right part), respectively.

9. Age-ranked profiles of the meta-measures of men and women

Figure S 10 shows profiles of selected characteristics of the participants and of their meta-measures stratified for gender and ranked with age. They show that body height and weight continuously decrease with age for both women and men. BMI, WHtR and ABSI steadily increase with age, however with a relatively high variance.

The body measures show increasing (e.g. A, B) and decreasing (H) mean courses with partially different levels (e.g. F) and different variabilities (e.g. C) for men and women. They hence reflect different dimensions of body shape.

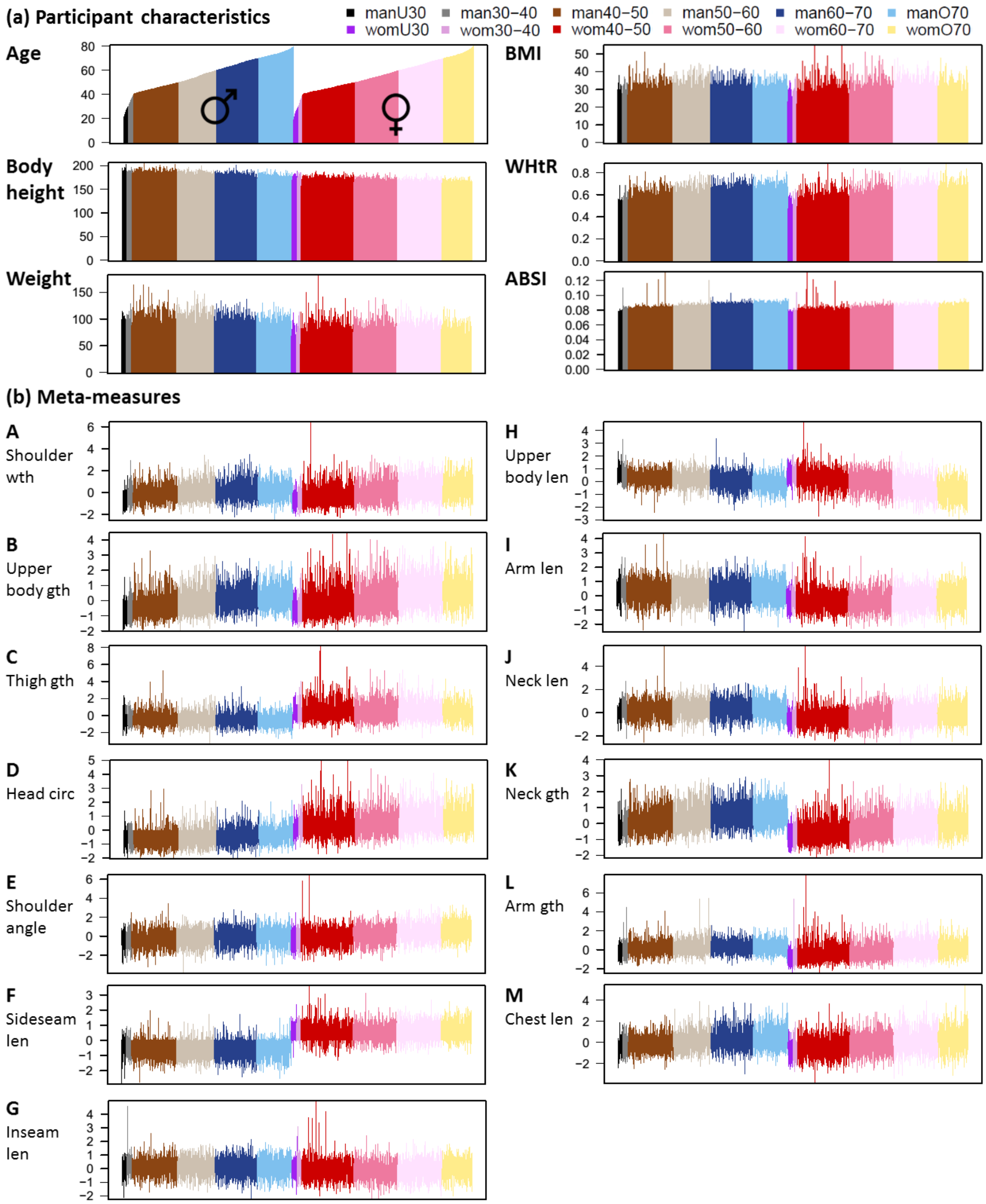

Figure S 10: Profiles of (a) selected participant characteristics and (b) meta-measures. Each bar represents one participant and is colored according to gender and age (see legend).

10. References

1. Wirth H, Löffler M, von Bergen M, Binder H: **Expression cartography of human tissues using self organizing maps**. *BMC Bioinformatics* 2011, **12**:306–352.

2. Vesanto J: **SOM-based data visualization methods**. *Intell data Anal* 1999, **3**:111–126.

3. Monti S, Tamayo P: **Consensus clustering: a resampling-based method for class discovery and visualization of gene expression microarray data**. *Mach Learn* 2003, **52**:91–118.
